# Promoting Empathy in Audiology Education Through Virtual Reality and Tactile Technologies: A Pilot Study for Patient-Centered Care in Individuals with Hearing Loss and Manual Dexterity Limitations

**DOI:** 10.1007/s40670-025-02403-x

**Published:** 2025-05-24

**Authors:** Razan Alfakir, Gracyn Holt, Lily Mason, Lily McGuckin, Yinbo Chen, Gary Hawkins, Liza Weisbrod, Aaron Trehub

**Affiliations:** 1 Department of Speech-Language & Hearing Sciences, Auburn University, Auburn, AL 36849 USA; 2 Auburn University Libraries Innovation and Research Commons (I&RC), Auburn, AL USA

**Keywords:** Hearing loss, Manual dexterity, Empathy, Virtual reality, Haptic glove

## Abstract

**Objective:**

This study evaluates the effectiveness of a virtual reality (VR)–based empathy training program, augmented with a haptic glove, in fostering cognitive, affective, and compassionate empathy among audiology students for patients with hearing loss (HL) and manual dexterity limitations.

**Methods:**

Using a mixed-methods design, two immersive VR simulation scenarios were developed to replicate challenges faced by patients with HL and impaired manual dexterity. Students participated in these scenarios while wearing Cambridge simulation gloves to restrict hand movement, simulating physical limitations. Pre- and post-intervention surveys assessed empathy using the Jefferson Scale of Empathy, alongside measures of immersion, usefulness, educational value, and adverse effects. Qualitative feedback provided insights into participants’ reflections.

**Results:**

Empathy scores significantly improved from pre- to post-intervention (Hedges’ *g* = 0.50, 95% CI 0.061–0.915; *P* < .05). High levels of immersion (74–86%), perceived usefulness (87%), and perceived educational value (91–100%) were reported. Qualitative feedback highlighted the training’s ability to engage students in the three key empathy dimensions.

**Conclusion:**

The VR-based training, integrated with haptic Cambridge simulation gloves, effectively enhanced students’ understanding of patient challenges related to HL and impaired manual dexterity. This innovative approach demonstrates the potential for fostering empathy in audiology education.

**Supplementary Information:**

The online version contains supplementary material available at 10.1007/s40670-025-02403-x.

## Introduction

Hearing loss (HL) is a prevalent chronic condition affecting approximately 30% of individuals over age 60 [1], with hearing aids being the most utilized intervention [2]. However, the successful use of hearing aids requires refined hand movements to manage small controls, insert and remove devices, and perform routine maintenance tasks. These actions demand a high degree of precision and dexterity — capabilities that often diminish with age.

Research highlights that adults over 60 frequently encounter reductions in hand function, limiting their ability to perform daily activities that require both fine and gross motor skills, such as fastening buttons, tying shoelaces, and managing small objects [3]. In addition to age-related decline, conditions such as osteoarthritis, rheumatoid arthritis, carpal tunnel syndrome, and peripheral neuropathy further impact hand functionality, complicating tasks that require fine motor skills [4–9]. Consequently, many patients with HL encounter substantial difficulty with hearing aid management tasks like adjusting volume, changing batteries, and cleaning components, which can lead to significant frustration and reduced satisfaction with their devices [6, 10, 11].

Recognizing these challenges, the International Classification of Functioning, Disability, and Health (ICF) has incorporated *Fine Hand Use (d440)* and *Hand and Arm Use (d445)* into its Comprehensive Core Set for Hearing Loss (CCSHL). This integration ensures that audiologic rehabilitation expands its focus to encompass physical limitations that affect hearing-related outcomes such as hearing aid use [12–16]. Also, in the context of ICF-CCSHL, environmental factors – including healthcare professionals, such as audiologists, categorized under Health Professionals (e355) – can either facilitate or impede patient outcomes related to HL [1, 12–16]. An audiologist’s capacity to empathize with patients experiencing impaired manual dexterity is, therefore, crucial for providing comprehensive, patient-centered care.

Empathy is a multidimensional construct essential to patient-centered care, particularly in audiology, where understanding the patient’s lived experience is key to effective intervention. It comprises three interrelated components: *cognitive empathy* (the ability to understand another person’s perspective), *affective empathy* (the ability to emotionally resonate with another’s feelings), and *compassionate empathy* (the motivation to take action to alleviate another’s suffering) [17–19]. Hence, active empathy requires audiologists to not only cognitively grasp but also emotionally connect with their patients’ experiences and respond compassionately. Despite its critical role in healthcare, empathy levels among American college students have shown a steady decline over the past few decades [20], raising concerns about the preparedness of future healthcare professionals.

The Jefferson Scale of Empathy (JSE) is a widely used instrument developed to assess empathy in the context of medical and health education and practice [21, 22]). It is designed to measure three key components of empathy: perspective- taking (the ability to adopt the patient’s point of view), compassionate care (the emotional connection and concern for the well-being of patients), and walking in the patient’s shoes (the capacity to emotionally resonate with the patient’'s experience). The JSE has been validated in multiple studies and is considered an effective tool for evaluating empathy in health education and practice.

Traditionally, audiology training programs have relied on structured, didactic approaches to teach empathy, including textbook readings, classroom lectures, and practical assignments such as case-based discussions and reflective writing assignments [23, 24]. While these approaches effectively contribute to developing a cognitive understanding of patient experiences, they often fall short in eliciting the emotional engagement necessary for a deep empathetic connection [25, 26]. In contrast, emerging evidence supports the use of virtual reality (VR)–based training as a more immersive and impactful approach to empathy education. VR enables experiential learning that can engage both cognitive and affective domains of empathy more effectively than traditional methods [25, 26].

With the advent of immersive technologies, VR has gained recognition as a promising tool in healthcare education [27–33]. According to the Society for Simulation in Healthcare, VR-based simulations are “a technique that creates a situation or environment to allow persons to experience a representation of a real event for practice, learning, evaluation, testing, or to gain an understanding of systems or human actions” [34]. These simulations allow learners to step into the patient’s world, offering firsthand insight into physical, emotional, and psychosocial challenges, especially for conditions like hearing loss, where many struggles are invisible. This immersive quality is particularly relevant when teaching students about patients with HL who also experience manual dexterity limitations, such as those affecting the use of hearing aids or assistive devices. VR allows students to experience the frustrations and barriers faced by these individuals in daily life, providing a level of empathy that traditional instruction often fails to cultivate. However, there is limited research on VR applications within audiology training.

This pilot study aimed to evaluate the effectiveness of a VR-based empathy training program, augmented with a haptic glove, in fostering cognitive, affective, and compassionate empathy among audiology students. We propose that VR simulations can help bridge the empathy gap by immersing students in realistic, patient-centered scenarios that reflect the complex, day-to-day challenges encountered by individuals with HL and reduced manual dexterity.

## Methods

### Participants and Recruitment

In the Fall of 2022, an interdisciplinary team at Auburn University, comprising faculty and trainees from the Computer Science and Software Engineering departments, along with library staff trained in 3D graphics and application design, developed and implemented EmpathyVR — an innovative activity designed to foster empathy for patients with restricted hand movement due to arthritis among student pharmacists [32]. Building on this foundation, the EmpathyVR project was expanded to include students enrolled in the Au.D. program at the Department of Speech, Language, and Hearing Sciences. This initiative aimed to deepen audiology students’ understanding of the challenges faced by patients with limited dexterity. The study protocol underwent a thorough review and received approval from the Institutional Review Board at Auburn University.

A total of 23 audiology students participated in the study, including 21 females and 2 males. Of the total, 15 were first-year Doctor of Audiology students, and 8 were in their second year of study. The mean age of the participants was 22.9 years (SD = 1.9). The majority of participants (21 out of 23) were white. Notably, A total of 17 students (74%) reported no prior exposure to empathy training, whereas 15 students (65%) acknowledged receiving minimal formal or informal instruction on the subject.

### Measures

Data was collected through anonymous surveys developed using Qualtrics. To preserve participant confidentiality while allowing for matched data analysis, surveys were linked using participant-generated identification codes. The survey included the following components:

- **Demographic information**: Participants provided details regarding gender identity, race, ethnicity, and age. In addition, they reported their prior exposure to VR experience, categorized as “never,” “once or twice,” or “three or more times.”
- **VR immersion (presence)**: Participants rated the degree of immersion experienced during the VR activity using the Nowak Presence Questionnaire, a 4-item modified version [35].
- **Perceived usefulness**: Participants rated their enjoyment of the VR activity using a 5-point Likert scale, ranging from “very enjoyable” to “not at all enjoyable.” The perceived usefulness of the activity in enhancing their understanding of patient needs and barriers to care was also assessed on a 5-point Likert scale, with responses ranging from “not at all useful” to “extremely useful.”
- **Perceived educational value:** Participants rated the perceived value of the VR activity using a 3-point Likert scale, ranging from “agree” to “disagree.”
- **Adverse effects**: Participants reported any adverse effects associated with the VR experience using the validated 9-item Virtual Reality Sickness Questionnaire [36, 37]. Symptom severity was rated on a 4-point scale, with options ranging from “not at all” to “very.”

To assess changes in empathy levels before and after the VR activity, the JSE-HPS was employed. The JSE-HPS consists of 20 items rated on a 7-point Likert scale, with total scores ranging from 50 to 140, where higher scores reflect greater empathy. Half of the items are positively worded and directly scored, and the other half are negatively worded (reverse scored).

#### Qualitative Feedback

At the end of the post-survey, participants were invited to provide qualitative feedback, offering additional insights into their personal experiences and perceptions of the VR activity.

### Development of VR Scenario

Two immersive VR scenarios were developed in collaboration with faculty from the Innovation and Research Commons (I&RC), located within Auburn University’s Ralph Brown Draughon Library. Each scenario was designed to simulate the lived experience of individuals with hearing loss and manual dexterity limitations in a realistic and emotionally engaging way. Each VR scenario lasted approximately 7–10 min. The first VR scenario simulated an audiology test booth, requiring students to press a button each time they heard a sound presentation. This activity enabled students to experience firsthand the challenges of finger control under physical limitations. The second scenario placed students in a room where various components of a hearing aid were arranged on a counter. In this setting, participants were instructed to pick up essential items for cleaning the hearing aid, such as a brush, dome, and battery. They were tasked with using the brush to clean the microphones, replacing the dome, and inserting the battery into the compartment.

### Procedures

The VR activity was conducted at Auburn University’s Ralph Brown Draughon Library. Upon arrival, students completed the informed consent process and filled out a pre-survey via Qualtrics, which included the JSE-HPS. To maintain anonymity, participant-generated codes were used throughout data collection. The students then engaged in the VR simulations. Each student was assisted by staff from the I&RC in donning the Meta Quest headset, which provided a fully immersive experience through a visually rich virtual environment. The headset utilized hand tracking to simulate natural hand movements, allowing students to interact with virtual objects. To simulate impaired manual dexterity, participants first wore Cambridge simulation gloves designed to restrict fine motor movements, followed by a second pair of gloves to enhance the hand-tracking capabilities of the VR system. Immediately after completing both VR scenarios, participants were asked to complete a post-survey, including the JSE-HPS. Before leaving the activity site, all participants took part in a 10-min facilitated debrief session to monitor physical side effects such as nausea or dizziness. Participants reporting symptoms were instructed to remain seated until they felt better.

### Data Analysis

Descriptive statistics were employed to characterize the sample and evaluate key variables, including presence, perceived usefulness, enjoyment, and adverse effects associated with the VR experience. To assess the impact of the VR activity on empathy levels, paired sample *t*-tests were conducted to compare pre- and post-questionnaire total scores on the JSE-HPS. Qualitative data collected from open-ended feedback were analyzed using thematic analysis to identify recurring patterns and themes among the students’ experiences.

## Results

### Quantitative Data

**Fig. 1:**
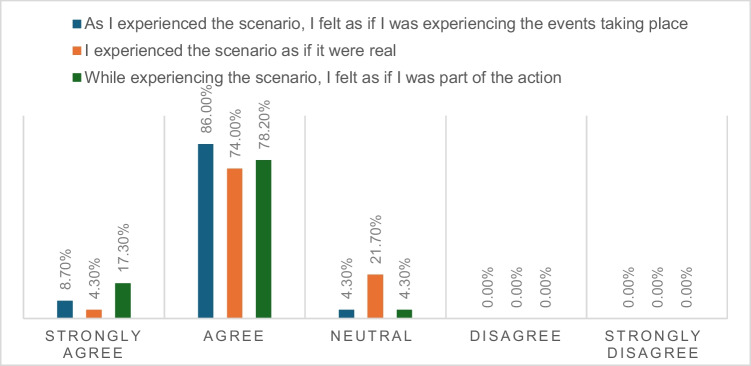
Presence-related EmpathyVR experiences

**Fig. 2:**
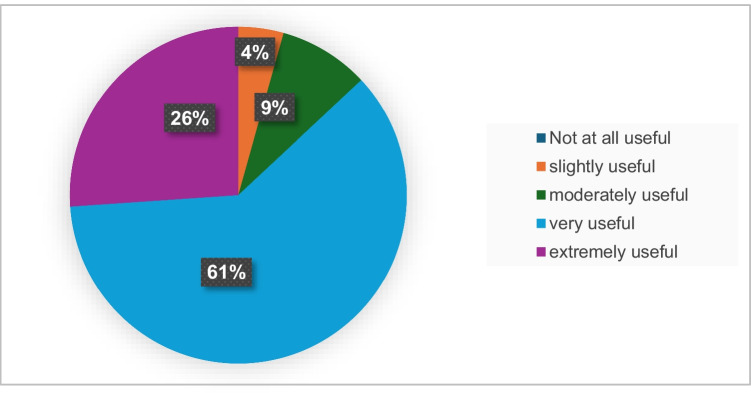
Perceived usefulness of the EmpathyVR training

**Fig. 3:**
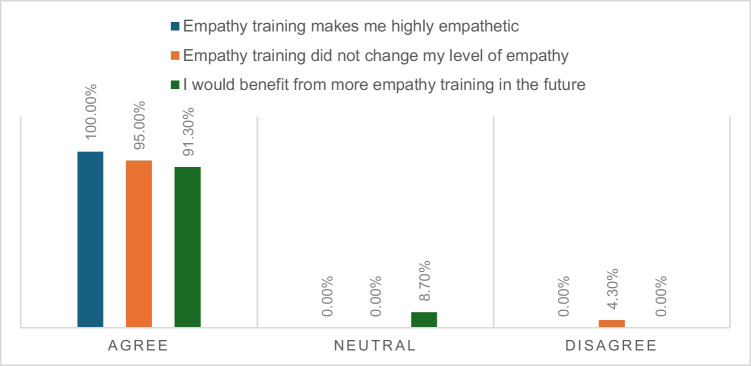
Perceived educational value of EmpathyVR experiences

Figures 1, 2, and 3 illustrate the perceived immersion (presence), usefulness, and educational value related to EmpathyVR experiences. Between 17 and 20 participants (74 to 86%) reported high levels of immersion (presence). Twenty participants (87%) perceived the activity as very to extremely useful, while between 21 and 23 participants (91 to 100%) indicated that it had educational value.

**Fig. 4:**
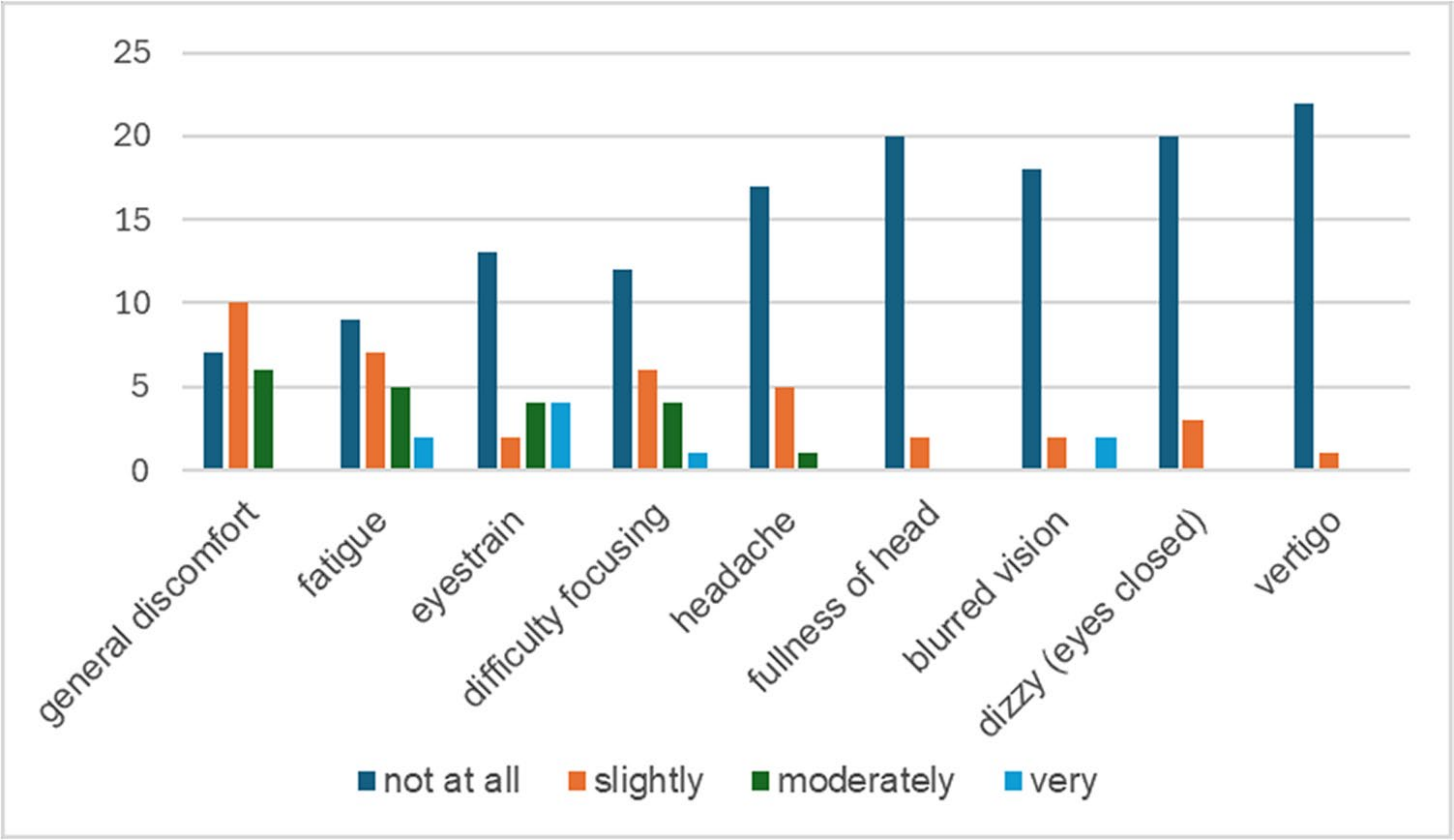
Virtual Reality Sickness Questionnaire (VRSQ)

Figure 4 presents the self-reported adverse effects associated with the EmpathyVR experience. General discomfort was reported by 16 participants (70%), and fatigue was noted by 14 participants (61%), with most rating these symptoms as “slight” or “moderate.” Notably, no participants reported experiencing nausea or dizziness following the VR activity. However, before departing the activity site, all participants engaged in a 10-min facilitated debriefing session.

Descriptive statistics for the JSE-HPS scores were calculated for both the pre- and post-intervention assessments. The pre-test scores ranged from 78.00 to 97.00, with a mean score of 86.09 (SD = 5.38). Post-test JSE Scores: The post-test scores ranged from 80.00 to 97.00, with a mean score of 88.74 (SD = 5.03). The data were further analyzed to examine the change in empathy scores from pre- to post-intervention. A paired samples *t*-test revealed a statistically significant increase in empathy scores from the pre-test (*M* = 86.09) to the post-test (*M* = 88.74), with a moderate effect size (Hedges’ *g* = 0.50; 95% CI, 0.061–0.915; *P* < 0.05).

**Fig. 5:**
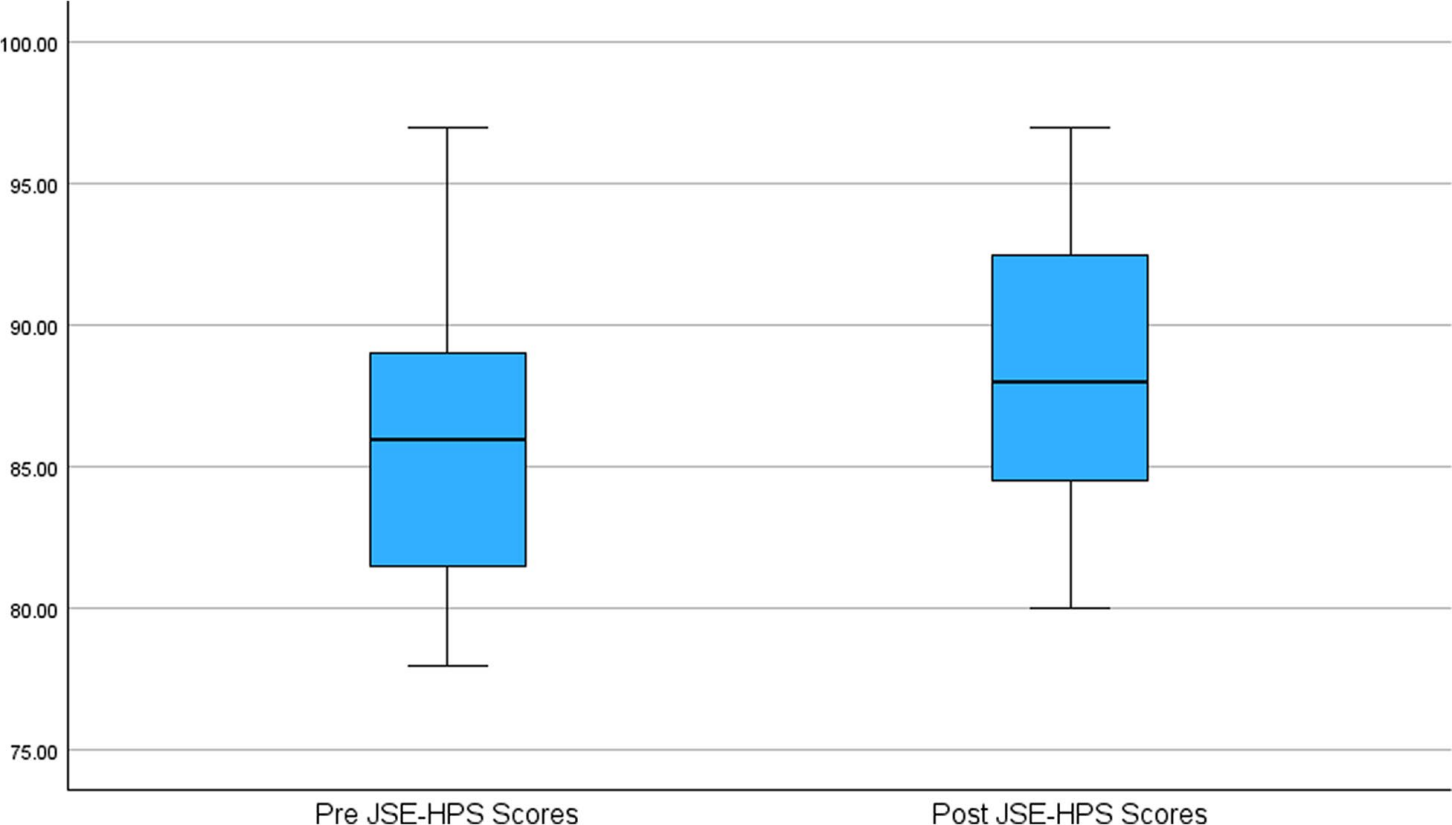
Comparison of pre and post JSE-HPS scores

The analysis of means, standard deviations, and *P*-values for each question on the JSE-HPS was not conducted, as the primary focus of this study was on the overall change in empathy levels. The JSE-HPS is intended to be interpreted as a composite measure; therefore, item-level analysis was not conducted, as it may not align with the scale’s psychometric design. Comparison of pre- and post-intervention among the JSE-HPS scores is presented in Fig. 5.

### Qualitative Data

Table 1 presents a thematic summary of qualitative feedback from participants, categorized according to domains of cognitive, affective, and compassionate empathy. Participants described a heightened awareness of the challenges encountered by individuals with limited dexterity, especially during routine activities such as manipulating hearing aids or responding to auditory stimuli. The VR experience facilitated both cognitive understanding and emotional resonance with these patient experiences, contributing to a deeper and more empathetic perspective. Moreover, many participants reported an increased sense of compassion and a strengthened intention to apply this empathy in future clinical practice. Several emphasized the pedagogical value of the activity and advocated for its inclusion in training programs for healthcare professionals.

**Table 1:** Participant feedback on EmpathyVR experience

Theme	Quote
**Cognitive and affective empathy** **Understanding empathy**	“The limited movement of my hands was more apparent when cleaning the HAs as opposed to pressing the button in response to the sound.” “Experiencing limited dexterity made me gain more empathy towards patients who may be experiencing the same or similar symptoms.” “I thought it was very helpful to experience troubles that a patient may be experiencing.”
**Compassionate empathy**	“This activity gave me a new perspective on patients with arthritis, and I can better empathize with them in the future.” “I think this training should be encouraged for all healthcare professionals.”

## Discussion

This study evaluates the effectiveness of a VR-based empathy training program, augmented with a Cambridge simulation gloves, in fostering cognitive, affective, and compassionate Empathy among audiology students for patients with HL and manual dexterity limitations. The findings of this study underscore the significant potential of immersive VR-based empathy training in enhancing students’ empathetic abilities, particularly across cognitive, affective, and compassionate dimensions. The high levels of immersion, perceived usefulness, and perceived educational value reflect the effectiveness of the VR platform in creating an engaging and impactful learning environment. These findings align with existing literature on VR’s capacity to simulate realistic scenarios that foster empathy and a deeper understanding of others’ perspectives [27–33].

### Multidimensional Nature of Empathy

Remarkably, empathy is a multifaceted construct comprising cognitive, affective, and compassionate dimensions [17]. Cognitive empathy, often referred to as perspective-taking, allows healthcare providers to intellectually comprehend patients’ experiences without necessarily sharing their emotions [38]. By promoting this skill, the program enhanced students’ ability to recognize and respect the challenges faced by patients with HL and manual limitations, fostering greater understanding and appreciation of their lived experiences. Beyond intellectual comprehension, affective empathy engages emotional resonance, enabling healthcare providers to feel with the patient. This emotional connection is critical for building trust, comfort, and rapport, ultimately enhancing the patient-provider relationship [23]. Compassionate Empathy motivates actionable responses to alleviate patients’ distress. The VR program effectively engaged this critical component, empowering students to translate empathy into meaningful support and intervention strategies, a hallmark of patient-centered care [23, 39]. The qualitative feedback substantiates these outcomes, with students reporting enhanced appreciation for patients’ struggles and an increased willingness to take compassionate actions to address their needs.

### Empirical Evidence of Impact

The program’s efficacy was further demonstrated through a statistically significant increase in empathy scores post-intervention, with a moderate effect size (Hedges’ *g* = 0.50; *P* < 0.05). This effect size underscores the meaningful impact of the intervention, positioning VR as a robust alternative to traditional empathy training methods. Importantly, adding the haptic glove likely played a critical role in heightening the realism and sensory engagement of the experience, which may have contributed to the notable outcomes.

### Challenges and Adverse Effects

While the results are promising, the study acknowledges some challenges. Participants reported adverse effects, including general discomfort and fatigue. These side effects, although not severe, highlight the need for optimizing VR hardware and intervention protocols. The absence of significant issues, such as nausea or dizziness, indicates the VR system’s relative tolerability. Future iterations could mitigate these minor discomforts by incorporating ergonomic improvements, shorter session durations, and regular breaks to enhance user comfort and reduce physical strain.

### Implications for Healthcare Education

This study makes a significant contribution to the field of healthcare education by demonstrating the transformative potential of immersive VR technology in fostering empathy. The program not only engaged students across all three dimensions of empathy but also equipped them with critical skills to improve patient outcomes. As healthcare increasingly prioritizes patient-centered care, tools like VR have the potential to revolutionize how empathy and interpersonal skills are taught, ensuring that future providers are better prepared to meet the complex needs of diverse patient populations.

### Future Directions

While the findings of this study are robust, further research is necessary to address several key areas. First, exploring the long-term effects of VR-based empathy training on clinical practice could provide valuable insights into its sustained impact on patient care. Second, assessing the scalability and cost-effectiveness of implementing such programs more broadly across healthcare curricula will be critical for widespread adoption. Third, investigating the adaptation of VR-based empathy training across diverse disciplines, including both healthcare and non-healthcare fields, can reveal its versatility and broader applicability. Lastly, integrating advanced technologies, such as AI-enhanced simulations, holds the potential to further personalize and optimize the training experience, ensuring its relevance and effectiveness for future learners.

### Limitations

A primary limitation of our study is the lack of a direct comparison between the effectiveness of VR-based learning scenarios and traditional formal learning methods, such as textbooks or assignments. This comparison is crucial for fully understanding the practical utility of VR as a pedagogical tool in audiology education. Without this comparative analysis, it is challenging to ascertain whether the immersive experiences provided by VR offer unique benefits over standard educational approaches. Future studies should incorporate a control group engaging in conventional learning methods to evaluate the distinct advantages of VR scenarios. Such research could clarify the added value of VR in fostering empathy and enhancing educational outcomes in healthcare training. Additionally, exploring various demographic factors, such as prior experience with technology or different learning styles, could provide further insights into how diverse student populations respond to VR-based learning.

Another limitation is the relatively small sample size, which may affect the generalizability of the findings. A larger, more diverse participant pool could yield more robust data and help identify trends that may not be evident in a smaller group. Furthermore, while the study focused on empathy development, it did not assess the long-term retention of empathetic skills or their application in real-world clinical settings. Future research could benefit from longitudinal studies that track the impact of VR training on empathy over time and in practice.

Lastly, while participants reported high levels of enjoyment and perceived usefulness, self-reported measures can be subjective. Incorporating objective assessments of empathy, such as the use of functional MRI, in future studies could provide a more comprehensive evaluation of the effectiveness of VR training [40]. By addressing these limitations in future research, we can better understand the role of VR in audiology education and its potential to transform how empathy is cultivated among healthcare providers.

### Conclusion

By combining immersive VR technology with pedagogical innovation, this study demonstrates the viability and effectiveness of empathy training programs that go beyond traditional methodologies. The significant improvements in empathy scores, coupled with high levels of user engagement, provide a strong foundation for integrating VR-based interventions into educational frameworks. As healthcare education evolves, such programs have the potential to bridge the gap between clinical competence and emotional intelligence, ultimately contributing to better patient care and outcomes.

## Supplementary Information

Below is the link to the electronic supplementary material.

ESM 1 (DOCX. 26 KB)

ESM 2 (DOCX. 30 KB

## Data Availability

The datasets generated and analyzed during the current study are available from the corresponding author upon reasonable request.
