# Supplementary material for: Promoting Empathy in Audiology Education Through Virtual Reality and Tactile Technologies: A Pilot Study for Patient-Centered Care in Individuals with Hearing Loss and Manual Dexterity Limitations
Source: Med Sci Educ. 2025 May 24;35(4):2011–9. doi: 10.1007/s40670-025-02403-x (PMC12532482; doi:10.1007/s40670-025-02403-x)
Supplement: Supplementary file 1 — (DOCX. 26 KB) [file 40670_2025_2403_MOESM1_ESM.docx]

**Participant #_______** **Date/Time________________**

**Please answer the following questions regarding demographics:**

**Q1.** **Gender**: How do you describe yourself?

- Male
- Female
- Non-binary/third gender
- Prefer not to say.
- Prefer to self-describe: __________________________________

**Q2. Age:** What is your age in years? _______________

**Q3. Ethnicity:** Are you Hispanic, Latino, or of Spanish origin?

- Yes
- No

**Q4. Race:** Choose one or more races that you consider yourself to be.

- White or Caucasian
- Black or African American
- American Indian/Native American or Alaska Native
- Asian
- Native Hawaiian or Other Pacific Islander
- Other
- Prefer not to say.

**"Please answer the following questions regarding your knowledge of chronic conditions like osteoarthritis, rheumatoid arthritis, carpal tunnel syndrome, and peripheral neuropathy, and the extent to which these conditions may impact hand functionality and complicated tasks requiring fine motor skills."**

**Q5.** Do you possess any information about how the above conditions affect the well-being and disability of patients seeking audiology care?

- not
- Definitely yes

**Q6.** Can you estimate to what extent the physical limitations in individuals with such a condition can influence their audiologic results?

- Minor influence
- Limited influence
- Moderate influence
- Major influence
- Overwhelming influence

Promoting empathy for patients with such chronic conditions via virtual reality and Tactile Technologies is an innovative training approach. **Please answer the following questions regarding your experience with empathy**:

**Q7**. Have you experienced empathy training before?

- Yes
- No

**Q8. If yes,** how has that empathy training benefited your patient-centered care?

**Q9.** **JSE-HPS Pre**- As a future audiologist, rate your agreement with the following statements about empathy.

1 — 2 — 3 — 4 — 5 — 6 — 7

Strongly disagree Strongly agree

| Statements | 1 | 2 | 3 | 4 | 5 | 6 | 7 |
| --- | --- | --- | --- | --- | --- | --- | --- |
| 1. My understanding of how my patients and their families feel does not influence medical or surgical treatment. |  |  |  |  |  |  |  |
| 1. My patients feel better when I understand their feelings. |  |  |  |  |  |  |  |
| 3. It is difficult for me to view things from my patients’ perspectives. |  |  |  |  |  |  |  |
| 4. I consider understanding my patients’ body language as important as verbal communication in caregiver-patient relationships |  |  |  |  |  |  |  |
| 5. I have a good sense of humor that I think contributes to a better clinical outcome. |  |  |  |  |  |  |  |
| 6. Because people are different, it is difficult for me to see things from my patients’ perspectives. |  |  |  |  |  |  |  |
| 7. I try not to pay attention to my patients’ emotions in history taking or in asking about their physical health. |  |  |  |  |  |  |  |
| 8. Attentiveness to my patients’ personal experiences does not influence treatment outcomes. |  |  |  |  |  |  |  |
| 9. I try to imagine myself in my patients’ shoes when providing care to them. |  |  |  |  |  |  |  |
| 10. My patients value my understanding of their feelings, which is therapeutic in its own right |  |  |  |  |  |  |  |
| 11. Patient’s illnesses can be cured only by medical or surgical treatment; therefore, emotional ties to my patients do not have a significant influence on medical or surgical outcomes. |  |  |  |  |  |  |  |
| 12. Asking patients about what is happening in their personal lives is unhelpful in understanding their physical complaints. |  |  |  |  |  |  |  |
| 13. I try to understand what is going on in my patients’ minds by paying attention to their non-verbal cues and body language |  |  |  |  |  |  |  |
| 14. I believe that emotion has no place in the treatment of medical illness |  |  |  |  |  |  |  |
| 15. Empathy is a therapeutic skill without which treatment success is limited. |  |  |  |  |  |  |  |
| 16. An important component of the relationship with my patients is my understanding of their emotional status, as well as that of their families. |  |  |  |  |  |  |  |
| 17. I try to think like my patients to render better care. |  |  |  |  |  |  |  |
| 18. I do not allow myself to be influenced by strong personal bonds between my patients and their family members. |  |  |  |  |  |  |  |
| 19. I do not enjoy reading non-medical literature or the arts |  |  |  |  |  |  |  |
| 20. I believe that empathy is an important therapeutic factor in medical or surgical treatment |  |  |  |  |  |  |  |
| **Total score** |  | | | | | | |
