# Supplementary material for: Promoting Empathy in Audiology Education Through Virtual Reality and Tactile Technologies: A Pilot Study for Patient-Centered Care in Individuals with Hearing Loss and Manual Dexterity Limitations
Source: Med Sci Educ. 2025 May 24;35(4):2011–9. doi: 10.1007/s40670-025-02403-x (PMC12532482; doi:10.1007/s40670-025-02403-x)
Supplement: Supplementary file 2 — (DOCX. 30 KB [file 40670_2025_2403_MOESM2_ESM.docx]

Participant #__________ Date/Time________________

**Please answer the following questions regarding your experience with virtual reality.**

Q1: In the computer-generated world, I had a sense of “being there.”

- Fully Disagree
- Disagree
- Neutral
- Agree
- Fully Agree

Q2: Somehow, I felt that the virtual world surrounded me.

- Fully Disagree
- Disagree
- Neutral
- Agree
- Fully Agree

Q3: I felt like I was just perceiving pictures.

- Fully Disagree
- Disagree
- Neutral
- Agree
- Fully Agree

Q4: I did not feel present in the virtual space.

- Fully Disagree
- Disagree
- Neutral
- Agree
- Fully Agree

Q5: I had a sense of acting in the virtual space, rather than operating something from outside.

- Fully Disagree
- Disagree
- Neutral
- Agree
- Fully Agree

Q6: How aware were you of the real world surrounding you while navigating in the virtual world? *(e.g., sounds, room temperature, other people, etc.)*

- Extremely Aware
- Aware
- Moderately Aware
- Barely Aware
- Not At All Aware

Q7: I was not aware of my real environment.

- Fully Disagree
- Disagree
- Neutral
- Agree
- Fully Agree

Q8: I still paid attention to the real environment.

- Fully Disagree
- Disagree
- Neutral
- Agree
- Fully Agree

Q9: I was completely captivated by the virtual world.

- Fully Disagree
- Disagree
- Neutral
- Agree
- Fully Agree

Q10: How did the virtual world seem to you?

- Completely Real
- Somewhat Real
- Neutral
- Barely Real
- Not At All Real

Q11: How much did your experience in the virtual environment seem consistent with your real-world experience?

- Not Consistent
- Barely Consistent
- Moderately Consistent
- Consistent
- Very Consistent

Q12: Please indicate your level of agreement with the statements below.

|  | Strongly Disagree | Disagree | Neutral | Agree | Strongly Agree |
| --- | --- | --- | --- | --- | --- |
| I was stressed when I tried to complete the task. |  |  |  |  |  |
| I felt physical discomfort while completing the tasks. |  |  |  |  |  |
| It seemed as though the touch I felt was caused by the hearing aid. |  |  |  |  |  |

Q13: Please indicate the extent to which you experienced the following symptoms while performing the activity.

|  | Not At All | Slightly | Moderately | Very |
| --- | --- | --- | --- | --- |
| General Discomfort |  |  |  |  |
| Fatigue |  |  |  |  |
| Eyestrain |  |  |  |  |
| Difficulty Focusing |  |  |  |  |
| Headache |  |  |  |  |
| Fullness of Head |  |  |  |  |
| Blurred Vision |  |  |  |  |
| Dizzy (eyes closed) |  |  |  |  |
| Vertigo |  |  |  |  |

**Please answer the following questions regarding your experience with the empathy training activity.**

Q14: Rate your level of mental engagement in the empathy learning activity.

- Very Engaged
- Somewhat Engaged
- Neutral
- Not Very Engaged
- Not At All Engaged

Q15: Rate the level of usefulness of the activity to support your learning about patient needs and barriers to hearing care.

- Not At All Useful
- Slightly Useful
- Moderately Useful
- Very Useful
- Extremely Useful

Q16: How would you express your experience with the training in empathy exercise if you see an audiology patient who has a medical history of a chronic condition like Rheumatoid Arthritis, for example?

|  | Agree | Neutral | Disagree |
| --- | --- | --- | --- |
| Empathy training makes me highly empathetic. |  |  |  |
| Empathy training did not change my level of empathy. |  |  |  |
| I would benefit from more empathy training in the future. |  |  |  |

Q17. JSE-HPS Pre - As a future audiologist, rate your agreement with the following statements about empathy after the empathy learning activity.

1 — 2 — 3 — 4 — 5 — 6 — 7

Strongly disagree Strongly agree

| Statements | 1 | 2 | 3 | 4 | 5 | 6 | 7 |
| --- | --- | --- | --- | --- | --- | --- | --- |
| 1. My understanding of how my patients and their families feel does not influence medical or surgical treatment. |  |  |  |  |  |  |  |
| 1. My patients feel better when I understand their feelings. |  |  |  |  |  |  |  |
| 3. It is difficult for me to view things from my patients’ perspectives. |  |  |  |  |  |  |  |
| 4. I consider understanding my patients’ body language as important as verbal communication in caregiver-patient relationships |  |  |  |  |  |  |  |
| 5. I have a good sense of humor that I think contributes to a better clinical outcome. |  |  |  |  |  |  |  |
| 6. Because people are different, it is difficult for me to see things from my patients’ perspectives. |  |  |  |  |  |  |  |
| 7. I try not to pay attention to my patients’ emotions in history taking or in asking about their physical health. |  |  |  |  |  |  |  |
| 8. Attentiveness to my patients’ personal experiences does not influence treatment outcomes. |  |  |  |  |  |  |  |
| 9. I try to imagine myself in my patients’ shoes when providing care to them. |  |  |  |  |  |  |  |
| 10. My patients value my understanding of their feelings, which is therapeutic in its own right |  |  |  |  |  |  |  |
| 11. Patient’s illnesses can be cured only by medical or surgical treatment; therefore, emotional ties to my patients do not have a significant influence on medical or surgical outcomes. |  |  |  |  |  |  |  |
| 12. Asking patients about what is happening in their personal lives is unhelpful in understanding their physical complaints. |  |  |  |  |  |  |  |
| 13. I try to understand what is going on in my patients’ minds by paying attention to their non-verbal cues and body language |  |  |  |  |  |  |  |
| 14. I believe that emotion has no place in the treatment of medical illness |  |  |  |  |  |  |  |
| 15. Empathy is a therapeutic skill without which treatment success is limited. |  |  |  |  |  |  |  |
| 16. An important component of the relationship with my patients is my understanding of their emotional status, as well as that of their families. |  |  |  |  |  |  |  |
| 17. I try to think like my patients to render better care. |  |  |  |  |  |  |  |
| 18. I do not allow myself to be influenced by strong personal bonds between my patients and their family members. |  |  |  |  |  |  |  |
| 19. I do not enjoy reading non-medical literature or the arts |  |  |  |  |  |  |  |
| 20. I believe that empathy is an important therapeutic factor in medical or surgical treatment |  |  |  |  |  |  |  |
| Total score |  | | | | | | |

**Please share any thoughts or feedback you have about the empathy learning activity below.**
